# Prostaglandin E_2_ directly inhibits the conversion of inducible regulatory T cells through EP2 and EP4 receptors via antagonizing TGF‐β signalling

**DOI:** 10.1111/imm.13417

**Published:** 2021-10-01

**Authors:** Marie Goepp, Siobhan Crittenden, You Zhou, Adriano G Rossi, Shuh Narumiya, Chengcan Yao

**Affiliations:** ^1^ Centre for Inflammation Research, Queen’s Medical Research Institute, The University of Edinburgh Edinburgh UK; ^2^ Systems Immunity University Research Institute, and Division of Infection and Immunity Cardiff University Cardiff UK; ^3^ Alliance Laboratory for Advanced Medical Research and Department of Drug Discovery Medicine, Medical Innovation Center Kyoto University Graduate School of Medicine Kyoto Japan

**Keywords:** EP receptors, Foxp3, inflammation, prostaglandin E_2_, regulatory T cell, TGF‐β

## Abstract

Regulatory T (Treg) cells are essential for control of inflammatory processes by suppressing effector T‐cell functions. The actions of PGE_2_ on the development and function of Treg cells, particularly under inflammatory conditions, are debated. In this study, we employed pharmacological and genetic approaches to examine whether PGE_2_ had a direct action on T cells to modulate de novo differentiation of Treg cells. We found that TGF‐β‐induced Foxp3 expression and iTreg cell differentiation in vitro is markedly inhibited by PGE_2_, which was mediated by the receptors EP2 and EP4. Mechanistically, PGE_2_‐EP2/EP4 signalling interrupts TGF‐β signalling during iTreg differentiation. Moreover, EP4 deficiency in T cells impaired iTreg cell differentiation in vivo. Thus, our results demonstrate that PGE_2_ negatively regulates iTreg cell differentiation through a direct action on T cells, highlighting the potential for selectively targeting the PGE_2_‐EP2/EP4 pathway to control T cell‐mediated inflammation.

## Graphical abstract

We have found that the development of inducible Treg cells is inhibited by PGE_2_ through its receptors EP2/EP4 and direct down‐regulation of TGF‐β signalling. Our results suggest a role for the PGE_2_‐EP2/EP4 pathway in T cell‐mediated inflammation.

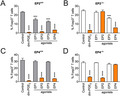

**Abbreviations**
cAMP: cyclic adenosine monophosphate
COX: cyclooxygenase
EP2: E prostanoid receptor 2
EP4: E prostanoid receptor 4
IL: interleukin
iTreg: inducible regulatory T cell
PG: prostaglandin
PGE_2_
: prostaglandin E_2_
TCR: T‐cell receptor
Teff: effector T cell
TGF‐β: transforming growth factor β
Treg: regulatory T cell

## INTRODUCTION

Regulatory T cells (Tregs) are a subset of T lymphocytes that play essential roles not only in the maintenance of immune homeostasis but also in the control of inflammatory responses [1, 2]. Treg cells actively suppress immune responses against autologous and foreign antigens in vitro and in vivo. Evidence from mouse models and human diseases indicates that eliminating Treg cell numbers or abrogation of their functions leads to a variety of immune‐mediated pathologies, including autoimmunity (e.g., multiple sclerosis, active rheumatoid arthritis and type 1 diabetes), allergies and graft rejection [3, 4, 5, 6, 7, 8]. Treg cells are characterized as expression of the surface marker CD25 (i.e., IL‐2 receptor α chain, IL‐2Rα) and the master transcription factor Forkhead box P3 (Foxp3) and produce the anti‐inflammatory cytokine IL‐10 [1]. Foxp3 controls both Treg cell development and their unique suppressive function [9, 10, 11]. Loss or mutation of Foxp3 expression links to a defective development of CD4^+^CD25^+^ Treg cells and in turn results in fatal autoimmune and inflammatory diseases, inducing a lymphoproliferative disorder in mice and leading to the IPEX (immunodysregulation, polyendocrinopathy, enteropathy, X‐linked) syndrome in human [12, 13].

There are two main subgroups of Treg cells in the body: natural (nTreg) and inducible Treg (iTreg) cells. Natural Treg cells arise in the thymus and can migrate into secondary lymphoid organs (spleen, lymph nodes, etc.). In addition, iTreg cells can be developed in the periphery by conversion from naïve Foxp3^–^ T effector (Teff) cells. The cytokine transforming growth factor β (TGF‐β) is a regulatory cytokine with an essential role in immune responses as well as in T‐cell tolerance [14, 15]. TGF‐β has both a direct role in regulating T effector cell differentiation, proliferation and apoptosis and an indirect role in the maintenance of immune homeostasis [16, 17]. It has been well documented that TGF‐β is required not only for the maintenance of the suppressive function and Foxp3 expression in nTregs but also for induction of Foxp3 expression in naïve CD4^+^ T cells and convert these cells into iTregs with a regulatory phenotype [18, 19, 20]. Lack or blockade of TGF‐β signalling reduces Treg cell numbers and impairs suppressive functions, leading to the development of autoimmune diseases [21]. Despite their critical roles in modulation inflammation, how the conversion of iTreg cells is controlled, especially by inflammatory mediators, is incompletely understood.

Prostaglandins (PGs) are a family of bioactive lipid mediators that are generated from arachidonic acid via the activities of cyclooxygenases (COXs) and selective PG synthases [22]. PGs, including PGE_2_, PGD_2_, PGF_2α_, PGI_2_ and thromboxane A_2_, play essential roles in numerous physiological and pathophysiological processes through autocrine and/or paracrine manners. Among PGs, PGE_2_ is found in the highest amounts in most tissues and is best studied. PGE_2_ has diverse effects on the development, regulation and activity of T cells through binding to its distinct G protein‐coupled receptors (called EP1‐4) [22]. For example, PGE_2_ inhibits T cell receptor (TCR) signalling, activation and then reduces production of cytokines such as IL‐2 and IFN‐γ through the EP2/EP4‐dependent cAMP‐PKA pathway [23]. However, PGE_2_ can also promote Th1 cell differentiation by inducing IL‐12Rβ1 expression through EP2/EP4‐dependent cAMP and PI3K signalling [24]. Moreover, PGE_2_ also fosters IL‐23‐dependent Th17 cell expansion and function by inducing IL‐23R expression through EP4/EP2 and the cAMP pathway [25, 26]. Importantly, emergent studies using pharmacological approaches and transgenic animal modelsthat target PGE_2_ receptors have demonstrated that the actions of PGE_2_ on T cells promote immune‐associated chronic inflammatory diseases in rodents and humans (including multiple sclerosis, rheumatoid arthritis, inflammatory skin and gut inflammation) [24, 25, 26, 27, 28, 29, 30]. While PGE_2_ was initially described to facilitate iTreg cell differentiation in vitro [31], it has also been reported to inhibit Foxp3 induction and reduce Treg cell numbers [32, 33, 34]. We have recently reported a T cell‐independent function of PGE_2_ on facilitation of Foxp3^+^ Treg cell responses in the intestine [35]. However, whether and how PGE_2_ directly influences iTreg cell differentiation remains to be elucidated.

In this study, we have employed pharmacological and genetic approaches to systemically examined the direct action of PGE_2_ in iTreg differentiation in vitro and in vivo using mice deficient in EP2 and EP4 receptors and highly selective small molecular reagents that target the respective PGE_2_ receptors. We found that PGE_2_ negatively regulated iTreg cell differentiation in vitro by inhibiting TGF‐β‐driven Foxp3 induction through EP2 and EP4. Lack of EP4 specifically in T cells increased Treg cell generation in vivo. The PGE_2_ pathway also appears to inhibit human iTreg cell differentiation. Our results have revealed that PGE_2_ directly acts on T cells to abrogate iTreg cell differentiation, which may contribute to foster T cell‐mediated inflammation.

## METHODS

### Animals

EP2^+/+^, EP2^–/–^ [36], EP4^+/+^, EP4^–/–^ [37], Lck^Cre^EP4^fl/fl^ [24, 38], *Rag*1^–/–^, Foxp3^YFP−Cre^ [39] and wild‐type C57BL/6 mice were bred and maintained under specific pathogen‐free conditions in accredited animal facilities at the University of Edinburgh and Kyoto University. Wild‐type mice were purchased from Harlan UK. Age‐ (>7 weeks old) and sex‐matched mice were used. Mice were randomly allocated into different groups and analysed individually. No mice were excluded from the analysis. All experiments were conducted in accordance with the UK Animals (Scientific Procedures) Act of 1986 with local ethical approval from the University of Edinburgh Animal Welfare and Ethical Review Body (AWERB) or approved by the Committee on Animal Research of Kyoto University Faculty of Medicine.

### Reagents and antibodies

16,16‐dimethyl prostaglandin E_2_ (dm‐PGE_2_) and PGE_2_ were purchased from Cayman Chemical. Highly selective agonists for EP1 (ONO‐DI‐004), EP2 (ONO‐AE1‐259–01), EP3 (ONO‐AE‐248) or EP4 (ONO‐AE1‐329) were gifts from Ono Pharmaceutical Co., Japan. Selective antagonists against EP2 (PF‐04418948) and EP4 (L‐161,982) were purchased from Cayman. Recombinant human TGF‐β1 and mouse or human IL‐2 were purchased from R&D system or Biolegend. Indomethacin, dibutyryl‐cAMP (db‐cAMP), 3‐isobutyl‐1‐methylxanthine (IBMX), H‐89, LY‐294002, AS1842856 and STAT5 inhibitor were purchased from Sigma or Calbiochem.

### T‐cell transfer

Naive CD4^+^CD25^−^CD62L^hi^ T cells were prepared from spleens of EP4^fl/fl^ or Lck^Cre^EP4^fl/fl^ mice by flow cytometry cell sorting. Cells (5 × 10^5^ cells per mouse) were transferred intravenously into Rag1^–/–^ mice. Mice were culled at 6 weeks after T‐cell transfer. Colons were collected for ex vivo analysis of lamina propria leucocytes.

### DSS application

Wild‐type C57BL/6 mice were given drinking water with dextran sulphate sodium (DSS, 2% w/v) or DSS plus indomethacin (5 mg per kg body weight per day) for 5 consecutive days before colons were collected for in vitro analysis of T cells.

### DNFB application

EP4^fl/fl^ and Lck^Cre^EP4^fl/fl^ mice were sensitized with 25 μl of 1% (w/v) Dinitrofluorobenzene (DNFB) in acetone/olive oil (4/1, v/v) on shaved abdominal skin on day 0. Skin‐draining lymph node cells were collected on day 5 for ex vivo analysis of T cells.

### T‐cell isolation and in vitro culture

Mouse CD4^+^CD25^−^ naïve T cells were isolated from spleens using Miltenyi Treg cell isolation kits. CD4^+^CD25^−^Foxp3(YFP)^−^ naïve T cells and CD4^+^CD25^+^Foxp3(YFP)^+^ nTreg cells were isolated from Foxp3^YFP−Cre^ mouse spleens by flow cytometry. Cells were cultured in complete RPMI1640 medium containing 10% FBS and stimulated with plate‐bound anti‐CD3 (5 μg/ml) and anti‐CD28 (5 μg/ml) antibodies plus various cytokines (IL‐2, rhTGF‐β1) and other compounds as indicated for 3 days. Human CD4^+^CD45RA^−^ naïve T cells were isolated from peripheral blood of healthy individuals, stimulated with plate‐bound anti‐CD3 and anti‐CD28, and then cultured with IL‐2 (10 ng/ml) and/or rhTGF‐β1 (10 ng/ml or indicated concentrations) for 3 days. PGE_2_ (1 μM or indicated concentrations) and its receptor agonists (1 μM) and other small molecular chemicals were added at the beginning of the culture or 24 hours later. Work with human blood cells was approved by the Centre for Inflammation Research (CIR) Blood Resource (AMREC Reference number 20‐HV‐069).

### Isolation of intestinal lamina propria leucocytes

Intestinal lamina propria cells were isolated as described previously [40].

### Staining and flow cytometry

For surface staining, cells were first stained with the Fixable Viability Dye eFluor® 780 (eBioscience) on ice for 30 min. After wash, cells were stained on ice for another 30 min with anti‐CD45 (clone 30‐F11), anti‐CD3e (Clone 145‐2C11), anti‐CD4 (Clone GK1·5) and anti‐CD25 (clone PC61·5). For staining of transcription factors, cells were fixed in the Foxp3/Transcription Factor Fix Buffer (eBioscience) for 2 h or overnight followed by staining with anti‐mouse Foxp3 (clone FJK‐16s), anti‐mouse Ki‐67 (clone 16A8) for at least 1 h. For cytokine staining, cells were restimulated ex vivo with PMA (50 ng/ml) and ionomycin (750 ng/ml) for 4 h in the presence of GolgiPlug (BD Bioscience), and then fixed and permeabilized following intracellular staining with anti‐mouse IL‐17A (clone ReBio17B7) and anti‐mouse IFN‐γ (clone RA3‐6B2) for 30 min. All Abs were purchased from eBioscience, Biolegends, or BD Bioscience. Flow cytometry was performed on the BD LSR Fortessa (BD Bioscience) and analysed by FlowJo software (Tree Star).

### Real‐time PCR

RNA purification from sorted MNPs was performed by using the RNeasy Mini Kit (Qiagen). cDNA was obtained by reverse transcription using the High‐capacity cDNA Reverse Transcription Kits (ABI). Samples were analysed by real‐time PCR with LightCycler Taqman Master (Roche) and Universal ProbeLibrary (UPL) Set, Mouse (Roche) on the LightCycler 2·0 (Roche). Primers were used are Glyceraldehyde‐3‐phosphate dehydrogenase (*Gapdh*) forward, 5’‐tgaacgggaagctcactgg‐3’; *Gapdh* reverse, 5’‐tccaccaccctgttgctgta‐3’. *Foxp3* forward: 5′‐cacccaggaaagacagcaacc‐3’; *Foxp3* reverse: 5′‐gcaagagctcttgtccattga‐3’. *Tgfbr1* forward: 5’‐aatgttacgccatgaaaatatcc‐3’; *Tgfbr1* reverse: 5’‐cgtccatgtcccattgtctt‐3’; UPL Probe: #84. *Tgfbr2* forward: 5’‐ggctctggtactctgggaaa‐3’; *Tgfbr2* reverse: 5’‐aatgggggctcgtaatcct‐3’; UPL Probe: #7. *Smad6* forward: 5’‐gttgcaacccctaccacttc‐3’; *Smad6* reverse: 5’‐ggaggagacagccgagaata‐3’; UPL Probe: #70. *Smad7* forward: 5’‐acccccatcaccttagtcg‐3’; *Smad7* reverse: 5’‐gaaaatccattgggtatctgga‐3’; UPL Probe: #63. Expression was normalized to *Gapdh* and presented as relative expression to the control group by the 2^–ΔΔCt^ method.

### Human gene expression analysis

We retrieved microarray data from Gene Expression Omnibus under an accession code (GSE71571) [41]. Raw data were normalized using the GC‐RMA method [42]. When multiple probe sets were present for a gene, the one with the largest variance was selected [43]. Change of the normalized expression levels for each gene by aspirin (i.e. aspirin–placebo) in colon biopsies was transformed into Z‐score, which was used to estimate the alteration of PGE_2_ pathway in each patient in response to Aspirin administration. The signature score of PGE_2_ pathway was estimated using a method described previously [44]. Briefly, we curated a gene list representative of PGE_2_ signature including its synthases and receptors. The final list consisted of *PTGS1*, *PTGS2*, *PTGES*, *PTGES2*, *PTGES3*, *PTGER2* and *PTGER4*. We weighted gene expression and computed a signature score per sample using singular‐value decomposition. Pearson's correlation coefficient was used to measure the association between PGE_2_ signature and expression of Treg genes on a Z‐score scale.

### Statistical analysis

Data were expressed as mean ±SEM, and statistical significance was performed by unpaired Student's *t* test or analysis of variance (ANOVA) with post hoc Holm‐Sidak's multiple comparisons test using Prism software (GraphPad). All *P* values <0.05 were considered as significant. Correlation analysis was calculated by Pearson's correlation coefficient (r).

## RESULTS

### PGE_2_ suppresses mouse iTreg differentiation in vitro

**FIGURE 1:**
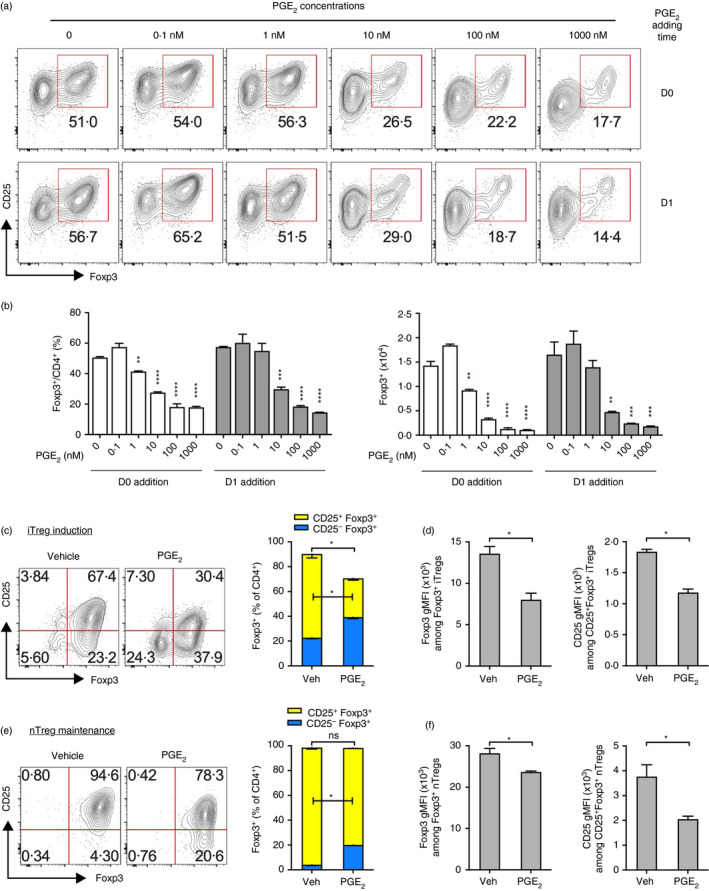
PGE_2_ suppresses iTreg cell differentiation in vitro. (a) Representative flow cytometry dot‐plot of CD25 and Foxp3 expression in CD4^+^CD25^−^ naïve T cells cultured under iTreg cell priming conditions (i.e. activated with anti‐CD3 and anti‐CD28 antibodies and stimulated with IL‐2 and TGF‐β1) from day 0 for 3 days. PGE_2_ was added with indicated concentrations and at indicated time‐points (i.e. day 0 or 1). (b) Accumulated percentages and numbers of Foxp3^+^ T cells. (c) Representative flow cytometry dot‐plot of CD25 and Foxp3 expression (left) and percentages of Foxp3^+^ T cells (right) in CD4^+^CD25^−^Foxp3(YFP)^−^ naïve T cells activated with anti‐CD3 and anti‐CD28 antibodies and cultured with IL‐2 and TGF‐β1 from day 0 for 3 days. PGE_2_ (100 nM) or vehicle control (Veh) was added to the cultures 1 d after TCR stimulation. (d) Geometric mean fluorescent intensity (gMFI) of Foxp3 and CD25 among Foxp3^+^ T cells. (e) Representative flow cytometry dot‐plot of CD25 and Foxp3 expression (left) and percentages of Foxp3^+^ T cells (right) in CD4^+^CD25^−^Foxp3(YFP)^+^ nTreg cells activated with anti‐CD3 and anti‐CD28 antibodies and cultured with IL‐2 and TGF‐β1 from day 0 for 3 days. PGE_2_ (100 nM) or vehicle control (Veh) was added to the cultures 1 d after TCR stimulation. (f) gMFI of Foxp3 and CD25 among Foxp3^+^ T cells. All experiments were performed in triplicates and repeated at least twice independently. *p < 0.05; **p < 0.01; ***p < 0.001; ****p < 0.0001. ns, not significant

We firstly examined whether PGE_2_ had an impact on iTreg differentiation in vitro. We isolated splenic CD4^+^CD25^−^ naïve T cells from wild‐type (WT) C57BL/6 mice, stimulated with anti‐CD3 and anti‐CD28 antibodies (Abs) and cultured with TGF‐β to induce the differentiation of iTreg cells. We added different concentrations of PGE_2_ (0 to 1000 nM) at the beginning of TCR stimulation on day 0. TGF‐β‐induced Foxp3 expression in CD4^+^ T cells was suppressed by addition of PGE_2_ in a concentration‐dependent manner (Figure 1a, b). To avoid PGE_2_ inhibition of TCR activation when it was added at the same time of anti‐CD3 stimulation [24], we tested the effect of PGE_2_ by postponing its time of addition to 24 h (day 1) after anti‐CD3 stimulation. Under this condition, PGE_2_ still inhibited TGF‐β‐induced Foxp3 expression (Figure 1a, b), suggesting that PGE_2_ prevents TGF‐β‐induced iTreg cell differentiation independently of its suppression on TCR activation.

A very small subpopulation of splenic CD4^+^CD25^−^ naïve T cells may express Foxp3. To examine whether the contamination of this small population of Foxp3^+^CD4^+^CD25^−^ T cells affects PGE_2_ inhibition on iTreg induction, we sorted splenic CD4^+^CD25^−^Foxp3(YFP)^−^ naïve T cells from Foxp3^YFP−Cre^ mice [39] and cultured them with TGF‐β. With this culture system, PGE_2_ still inhibited Foxp3 induction (Figure 1c). Interestingly, PGE_2_ specifically reduced CD25^+^Foxp3^+^ cells (Figure 1c), a Treg subpopulation that has greater immunosuppressive function compared to CD25^−^Foxp3^+^ Treg cells [21, 45]. Furthermore, PGE_2_ treatment reduces mean fluorescent intensity (MFI) of Foxp3 and CD25 (Figure 1d), suggesting that PGE_2_ also inhibits Foxp3 expression at the single cell level.

To examine whether PGE_2_ affects the stability of Foxp3 expression on nTreg cells, we sorted splenic CD4^+^CD25^+^Foxp3(YFP)^+^ nTreg cells from Foxp3^YFP−Cre^ mice and cultured with TGF‐β for 3 days. Addition of PGE_2_ did not affect total percentage of Foxp3^+^ cells, but appeared to reduce the MFI of Foxp3 (Figure 1e, f). Moreover, PGE_2_ treatment significantly reduced CD25 expression, leading to a reduction of the CD25^+^Foxp3^+^ nTreg subpopulation (Figure 1e, f). Taken together, these results suggest that PGE_2_ represses both de novo iTreg cell differentiation and, to a less extent, Treg maintenance.

### EP2 and EP4 receptors mediate PGE_2_ suppression of iTreg differentiation in vitro

**FIGURE 2:**
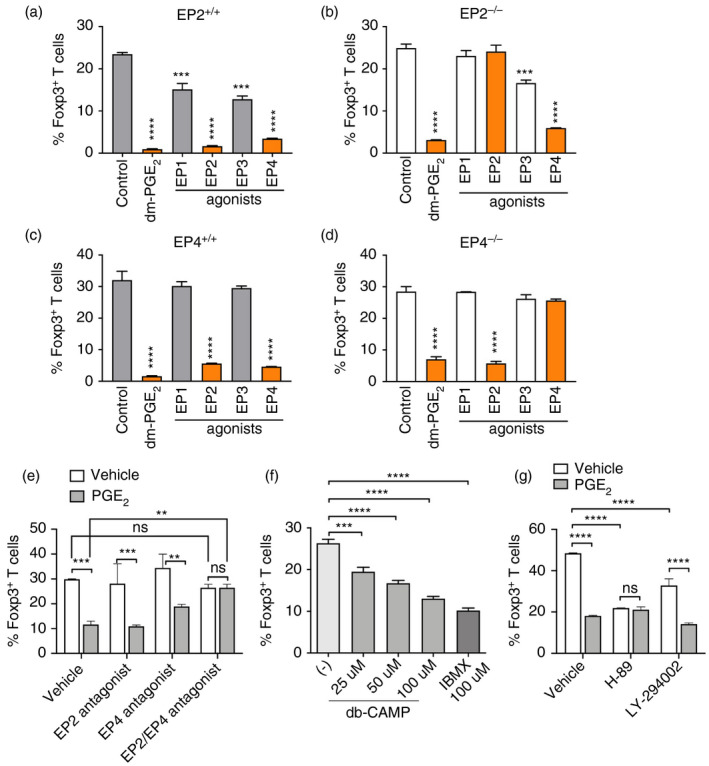
EP2 and EP4 receptors mediate PGE_2_ suppression of iTreg cell differentiation *in vitro*. (a,b) Percentages of Foxp3^+^ T cells in EP2^+/+^ (a) or EP2^−/−^ (b) CD4^+^CD25^−^ naïve T cells cultured with IL‐2 and TGF‐β1 with dm‐PGE_2_ or selective agonists for each EP1‐4 receptor for 3 days. (c,d) Percentages of Foxp3^+^ T cells in EP4^+/+^ (c) or EP4^−/−^ (d) CD4^+^CD25^−^ naïve T cells cultured with IL‐2 and TGF‐β1 with dm‐PGE_2_ or selective agonists for each EP1‐4 receptor for 3 days. (e) Percentages of Foxp3^+^ T cells in wild‐type C57BL/6 CD4^+^CD25^−^ naïve T cells cultured with IL‐2 and TGF‐β1 in the absence or presence of PGE_2_, EP2 antagonist or EP4 antagonist or both EP2 and EP4 antagonists for 3 days. (f) Percentages of Foxp3^+^ T cells in wild‐type C57BL/6 CD4^+^CD25^−^ naïve T cells cultured with IL‐2 and TGF‐β1 with db‐cAMP or IBMX for 3 days. (g) Percentages of Foxp3^+^ T cells in wild‐type C57BL/6 CD4^+^CD25^−^ naïve T cells cultured with IL‐2 and TGF‐β1 with PGE_2_, a PKA inhibitor (H‐89) or a PI3K inhibitor (LY‐294002) for 3 days. All experiments were performed in triplicates and repeated at least twice independently. *p < 0.05; **p < 0.01; ***p < 0.001; ****p < 0.0001. ns, not significant

Next, we investigated which PGE_2_ receptors mediated the suppression of iTreg differentiation. We isolated splenic CD4^+^ T cells from EP2‐ or EP4‐deficient and WT control mice, cultured with TGF‐β with the addition of dm‐PGE_2_ (a stable PGE_2_ analogue) or selective agonists for PGE_2_ receptors EP1‐EP4. In EP2^+/+^ (on the C57BL/6 genetic background) or EP4^+/+^ mice (on the mixed C57BL/6 x 129 genetic background), EP2 and EP4 agonists mimicked PGE_2_ suppression of TGF‐β‐induced Foxp3 expression from CD4^+^CD25^−^ naïve T cells (Figure 2a, c). Deficiency of EP2 or EP4 alone had no impact on TGF‐β‐induced Foxp3 expression from naïve T cells (Figure 2b, d), suggesting that EP2 and EP4 are not required for iTreg induction. This may be due to two possibilities –(1) naïve and TCR‐activated mouse T cells do not produce or produce very low levels of endogenous PGE_2_, and/or (2) endogenous PGE_2_‐EP2 and PGE_2_‐EP4 signalling have redundant effects on repression of iTreg induction. In EP2^−/−^ CD4^+^CD25^−^ naïve T cells, however, EP2 agonist failed to suppress Foxp3 expression although PGE_2_ and EP4 agonist still have inhibitory effects (Figure 2b). Likewise, EP4 agonist had no effect on induction of Foxp3 expression from EP4^−/−^ CD4^+^CD25^−^ naïve T cells, but PGE_2_ and EP2 agonist still repressed iTreg induction (Figure 2d). Selective EP1 and EP3 agonists appeared only mild inhibition of Foxp3 induction in C57BL/6 EP2^+/+^ T cells and had no influences on EP2^−/−^, EP4^+/+^ or EP4^−/−^ T cells (Figure 2a–d). Furthermore, inhibition of Foxp3 expression by PGE_2_ was rescued by combination of EP2 and EP4 antagonists, but not by blockade of either single receptor alone (Figure 2e). These results suggest that PGE_2_ suppression of iTreg cell differentiation in vitro is redundantly mediated by EP2 and EP4 receptors.

Given EP2 and EP4 activate the cyclic adenosine monophosphate (cAMP) and PI3K signalling pathways [22], we examined whether these pathways mediate the suppression of iTreg cell induction. We used dibutyryl cAMP (db‐cAMP, a cell‐permeable cAMP analogue) and isobutylmethylxanthine (IBMX, a phosphodiesterase inhibitor that blocks cAMP degradation) to increase the intracellular cAMP levels. Similar to PGE_2_, both db‐cAMP and IBMX prevented TGF‐β‐dependent conversion of Foxp3^+^ iTreg cell (Figure 2f). Blockade of the cAMP pathway by a PKA inhibitor (H‐89) or the PI3K pathway by LY‐294002 repressed TGF‐β‐dependent Foxp3 expression (Figure 2g). PGE_2_ had no additive suppression of Foxp3 induction with H‐89, but did further reduced Foxp3 expression in the presence of LY‐294002 (Figure 2g). These results indicate that the cAMP/PKA, rather than PI3K, pathway is involved in PGE_2_‐dependent inhibition of iTreg cell differentiation.

### PGE_2_ antagonizes TGF‐β signalling during iTreg differentiation

**FIGURE 3:**
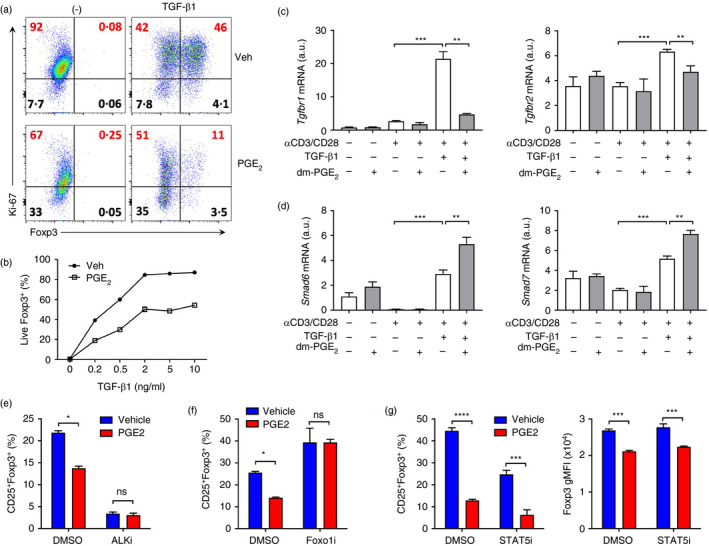
PGE_2_ antagonizes TGF‐β signalling during iTreg cell differentiation. (a) Representative flow cytometry dot‐plot of Foxp3 and Ki‐67 expression in CD4^+^CD25^−^ naïve T cells cultured with IL‐2 and TGF‐β1 in the absence or presence of PGE_2_ for 3 days. (b) Percentages of live Foxp3^+^ T cells in CD4^+^CD25^−^ naïve T cells cultured with IL‐2 and indicated concentrations of TGF‐β1 in the absence or presence of PGE_2_ for 3 days. (c,d) Expression of *Tgfbr1*, *Tgfbr2*, *Smad6* and *Smad7* genes in CD4^+^CD25^−^ naïve T cells cultured with or without anti‐CD3/CD28, TGF‐β1 or PGE_2_ for 3 days. (e‐g) Percentages of CD25^+^Foxp3^+^ T cells in CD4^+^CD25^−^ naïve T cells cultured with IL‐2 and TGF‐β1 in the absence or presence of PGE_2_ and inhibitors for ALK (ALKi, E), Foxo1 (Foxo1i, F) or STAT5 (STAT5i, G) for 3 days. Geometric mean fluorescent intensity (gMFI) of Foxp3 among Foxp3^+^ T cells (G). *p < 0.05; **p < 0.01; ***p < 0.001. ns, not significant

We next examined the mechanisms by which PGE_2_ inhibits iTreg cell differentiation. We stained T cells with Ki‐67, an intracellular marker of cell proliferation. In the absence of TGF‐β, PGE_2_ moderately prevented anti‐CD3/CD28‐induced naïve T‐cell proliferation, evidenced as Ki‐67^+^Foxp3^−^ T cells (Figure 3a). Under the iTreg cell differentiation condition, TGF‐β markedly induced and expanded Ki‐67^+^Foxp3^+^ proliferative iTreg cells (Figure 3a). However, this was significantly prevented by PGE_2_ which had few effects on Ki‐67^+^Foxp3^−^ non‐Treg cells (Figure 3a), indicating that PGE_2_ selectively prevents TGF‐β‐dependent induction of proliferating iTreg cells. Indeed, PGE_2_ suppressed TGF‐β responsiveness during Foxp3^+^ iTreg differentiation (Figure 3b).

During iTreg differentiation, TGF‐β firstly activates gene expression of its receptors (i.e. *Tgfbr1* and *Tgfbr2*) on T cells, which were both repressed by the addition of PGE_2_ (Figure 3c). TGF‐β also stimulates T cells to express Smad6 and Smad7 [46], endogenous inhibitors for TGF‐β signalling, which were significantly further upregulated by PGE_2_ (Figure 3d). These results suggest an inhibitory effect of PGE_2_ on TGF‐β signalling in T cells, as seen in other cell types [47, 48, 49]. To further study the possibility of PGE_2_ influence on TGF‐β signalling, we used a small molecular ALK inhibitor, which blocks the TGF‐β/TGF‐β receptor/Smad pathway. ALK inhibitor itself significantly repressed TGF‐β‐dependent iTreg cell induction, and addition of PGE_2_ had no additional effects on Foxp3 induction in the present of with the ALK inhibitor (Figure 3e). The transcription factor Foxo1 acts downstream of TGF‐β receptors, and is responsible for TGF‐β responsiveness in iTreg cell differentiation [50]. The Foxo1 inhibitor (AS1842856) did not affect TGF‐β‐dependent Foxp3 induction, but it reversed PGE_2_ suppression of Foxp3 induction (Figure 3f). These results suggest that PGE_2_ suppresses the process of iTreg differentiation by antagonizing TGF‐β signalling.

In response to TCR engagement, activated T cells produce large amount of IL‐2, which is also essential for iTreg cell differentiation through the transcription factor STAT5 [51, 52]. As PGE_2_ strongly inhibits TCR activation and IL‐2 production, we asked whether PGE_2_ suppresses iTreg cell induction via inhibiting IL‐2‐STAT5 signalling. We cultured T cells under the iTreg‐skewing condition and used a STAT5 inhibitor (STAT5i). As expected, the STAT5 inhibitor suppressed iTreg cell conversion compared to vehicle control (Figure 3g). However, PGE_2_ was still able to further down‐regulate Foxp3 expression in the presence of STAT5 inhibitor (Figure 3g). Thus, IL‐2‐STAT5 signalling is unlikely to be involved in PGE_2_ suppression of mouse iTreg cell induction.

### Lack of EP4 impairs iTreg cell differentiation in vivo

**FIGURE 4:**
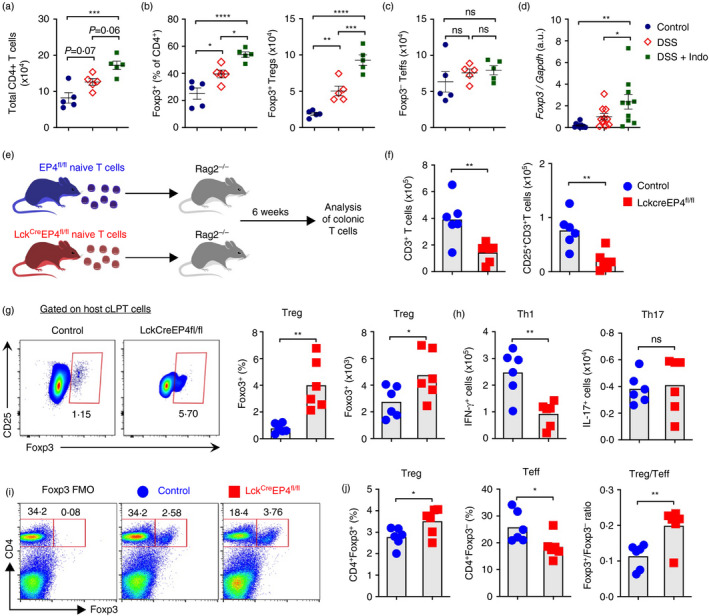
PGE_2_ represses Treg cell differentiation in vivo. (a) Total CD3^+^CD4^+^ T cells in colonic lamina propria of mice treated with vehicle or 2% DSS in drinking water or DSS plus indomethacin in drinking water for 5 days. (b) Percentages and numbers of colonic Foxp3^+^ Treg cells. (c) Numbers of colonic Foxp3^–^ Teff cells. (d) Foxp3 gene expression in whole colon tissues. (e) Schematic representation of the experimental protocol for T cells transfer. CD4^+^CD25^−^CD62L^hi^ naïve T cells isolated from LcK^Cre^EP4^fl/fl^ and control EP4^fl/fl^ mice were transferred into Rag1^−/−^ mice. Colonic lamina propria T cells in host Rag1^−/−^ mice were analysed 6 weeks later. (f) Numbers of colonic CD3^+^ total T cells and CD25^+^ activated T cells. (g) Representative flow cytometry dot‐plot of Foxp3 and CD25 expression, percentages and absolute numbers of Foxp3^+^ T cells in colons. (h) Absolute numbers of colonic Th1 and Th17 cells. (i) Representative flow cytometry dot‐plot of Foxp3 and CD4 expression in skin‐draining lymph nodes of LcK^Cre^EP4^fl/fl^ and control EP4^fl/fl^ mice that were sensitized with DNFB. (j) Percentages of Foxp3^+^ Treg and Foxp3^−^ T effector (Teff) cells and the ratio of Treg vs Teff cells in dLNs. Each dot represents one mouse. *p < 0.05; **p < 0.01; ***p < 0.001; ****p < 0.0001. ns, not significant

We have recently found that blockade of endogenous PGE_2_ production in naïve WT mice by inhibition of COX activities increased Foxp3^+^ Treg cell numbers in the intestine [35]. To examine whether blockade of endogenous PGE_2_ production also enhances Treg cell responses under inflammatory conditions, we used 2% dextran sulphate sodium (DSS) to induce acute colonic inflammation in WT C57BL/6 mice. DSS treatment increased accumulation of total T cells in the colon, which was further enhanced by co‐administration of indomethacin, a non‐selective COX inhibitor (Figure 4a). This is consistent with previous report that blocking COX activity exacerbated DSS‐dependent intestinal inflammation [40]. Interestingly, indomethacin also significantly increased numbers of Foxp3^+^ Treg cells, but not Foxp3^–^ Teff cells, in inflamed colons (Figure 4b, c), which was in line with upregulated Foxp3 gene expression in the colon tissues (Figure 4d). These results suggest that endogenous PG signalling represses Treg cell response under inflammatory conditions.

To further examine whether PGE_2_ signalling directly modulates Treg cell responses in vivo. We crossed EP4‐floxed mice to Lck‐Cre mice to generate T cell‐specific EP4 deficient mice (Lck^Cre^EP4^fl/fl^). Lck^Cre^EP4^fl/fl^ and control EP4^fl/fl^ mice had comparable nTregs in the thymus [35], suggesting that lack of EP4 signalling in T cells does not affect nTreg cell development in vivo. To examine whether PGE_2_ affects iTreg cell differentiation in vivo, we sorted naïve CD3^+^CD4^+^CD25^−^CD62L^+^ T cells from Lck^Cre^EP4^fl/fl^ and control EP4^fl/fl^ mice, and then transferred these cells into Rag1^−/−^ mice that have no T and B cells (Figure 4e). Upon transfer, naïve T cells are activated, proliferated and differentiated into T effect cells (e.g. Th1 and Th17 cells) in the host mice and accumulated in the large intestines. Simultaneously, a small population of naive T cells are differentiated into Foxp3^+^ iTreg cells. Lack of EP4 signalling reduced total T cells migration to the colon and down‐regulation of T‐cell activation evidenced by reduction of CD25 expression (Figure 4f). In contrast, differentiation of Foxp3^+^ Tregs in the host mouse colons from EP4‐deficient naive T cells was greater than that from control EP4‐sufficient naïve T cells (Figure 4g). In agreement with our previous findings [24], Rag1^−/−^ mice transferred with EP4‐deficient naïve T cells had less IFN‐γ^+^ Th1 cells compared to mice that were transferred with control naïve T cells, but EP4 deficiency had no influence on colonic IL‐17^+^ Th17 cells in the host mice (Figure 4h). To further confirm the effect of EP4 signalling on Treg responses in vivo, we sensitized Lck^Cre^EP4^fl/fl^ and control EP4^fl/fl^ mice with a hapten dinitrobenzfluorene (DNBF) on the abdominal skin and analysed T cells in skin‐draining lymph nodes. Again, lack of EP4 signalling in T cells significantly increased Foxp3^+^ Treg cells but reduced Foxp3^−^ effector T cells in draining lymph nodes (Figure 4i, j). Together, these results indicate that PGE_2_‐EP4 signalling directly acts on T cells to impede iTreg cell differentiation in vivo.

### Inhibition of human iTreg cell differentiation by PGE_2_

**FIGURE 5:**
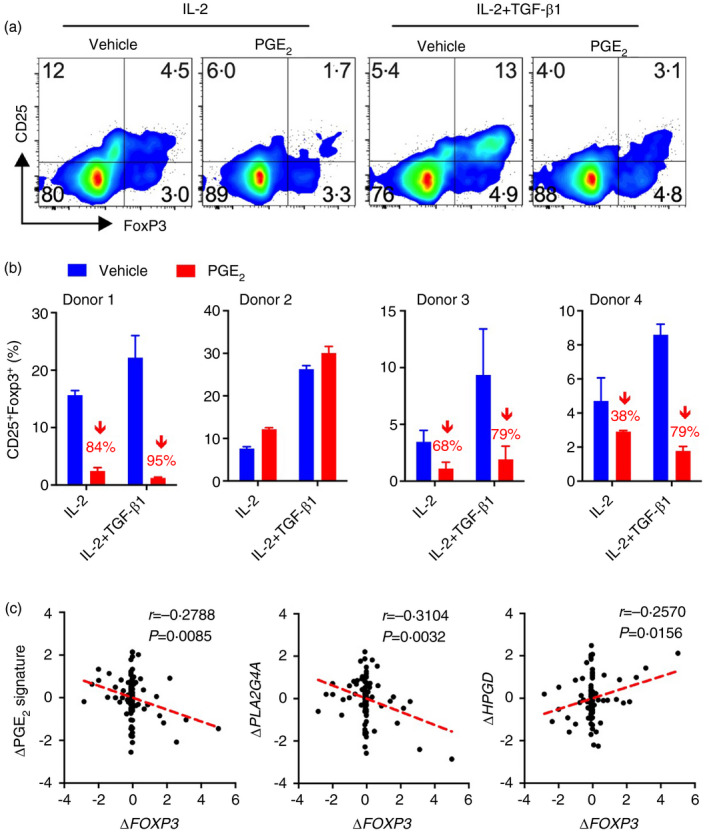
PGE_2_ inhibits human iTreg cell differentiation. (a) Representative flow cytometry dot‐plot of Foxp3 and CD25 expression in CD4^+^CD45RA^−^ naïve T cells that were isolated from healthy human blood, stimulated with anti‐CD3 and anti‐CD28, and cultured IL‐2 alone or IL‐2 + TGF‐β1 in the absence or presence of PGE_2_ for 3 days. (b) Accumulated percentages of CD25^+^Foxp3^+^ human iTreg cells from four individual donors. Numbers in red represent the efficiency of PGE_2_ inhibition of Foxp3 induction (i.e. down‐regulation in percentages of Vehicle). (c) Microarray gene expression data from human colon biopsies in response to aspirin administration for 2 months in healthy individuals were analysed for the association of the PGE_2_ pathway signature gene expression with that of Treg‐related genes. Correlations between the PGE_2_ signature scores or *HPGD* expression levels and *FOXP3* gene expression from total tested samples (*n* = 88). Raw gene expression data were retrieved from Gene Expression Omnibus GSE71571. Standardized expression values represent changes (Δ) of gene expression levels before and after aspirin treatment and then transformed to Z‐scores. Each dot represents one sample. Statistical analysis was calculated by two‐tailed Pearson correlation coefficients (*r*), and a linear regression‐fitting curve is shown as the red dotted line

To corroborate our findings from mouse T cells, we examined whether PGE_2_ suppresses human iTreg cell differentiation. We isolated CD4^+^CD45RA^−^ naïve T cells from peripheral blood of healthy individuals, stimulated with anti‐CD3 and anti‐CD28 Abs and cultured with IL‐2 alone or IL‐2 plus TGF‐β. Addition of PGE_2_ suppressed Foxp3 expression from 3 out of 4 donors in T‐cell cultures with not only IL‐2 plus TGF‐β but also IL‐2 alone by similar degree, albeit slight less suppression in the latter (Figure 5a, b), suggesting that PGE_2_ also inhibits human iTreg cell differentiation possibly through a different mechanism from that in mouse.

We then asked whether the expression levels of PGE_2_ signalling pathway genes were correlated with *FOXP3* gene expression in human tissues. We examined a public dataset from a clinical trial which measured gene expression of colon biopsies obtained from healthy individuals before and after administration of aspirin (325 mg/d, daily for 60 days) [41]. We correlated the changes in mRNA expression of PGE_2_ pathway signature genes (including PGE_2_ synthases: *PTGS1*, *PTGS2*, *PTGES*, *PTGES2*, *PTGES3* and receptors: *PTGER2*, *PTGER4*) before and after aspirin administration with changes in Foxp3 gene expression. Changes in PGE_2_ pathway genes by aspirin treatment were negatively correlated with changes in *FOXP3* gene expression (Figure 5c). COX‐mediated biosynthesis of PGs including PGE_2_ relies on the release of arachidonic acid from cellular phospholipids, which is mediated by the cytosolic phospholipase A2 (cPLA2, encoded by the *PLA2G4A* gene). Aspirin and other non‐steroidal anti‐inflammatory drugs inhibit PG production through not only blocking COX activities but also suppression of *PLA2G4A* gene expression and subsequently the substrate of COXs [53]. Similarly, changes in expression of the *PLA2G4A* gene before and after aspirin administration was also inversely correlated with changes in *FOXP3* gene expression (Figure 5c). In contrast, changes in expression of *HPGD* (which mediates the metabolic inactivation of PGE_2_ to 15‐keto PGE_2_) was positively correlated with changes in *FOXP3* gene expression (Figure 5c). These results suggest that changes in gene expression involved in PGE_2_ synthesis and signalling pathways is inversely associated with alteration of *FOXP3* gene expression in healthy human gut tissues.

## DISCUSSION

PGE_2_ was initially reported to induce Foxp3 expression and iTreg induction and enhance Treg suppressive function, therefore contributing to antitumour T‐cell responses [54, 55]. Indeed, positive correlations between COX2 and Foxp3 expression have been found in multiple tumour tissues [55, 56, 57]. PGE_2_ was thought to promote Treg cells through both direct actions on T cells [54] and indirect actions on other cell types such as dendritic cells or myeloid‐derived suppressor cells [58, 59, 60]. On the other side, PGE_2_ has also been reported to suppress Treg cell differentiation and signature gene (e.g. Foxp3, IL‐10) expression from both mouse and human effector T cells through direct actions on T cells via EP2 and/or EP4 receptors [32, 33, 61, 62, 63, 64]. In agreement with these reports, blocking PG biosynthesis including PGE_2_ production by NSAIDs or blocking PGE_2_ signalling by an EP4 selective antagonist enhanced Foxp3 expression and iTreg induction, and therefore ameliorated T cell‐mediated tissue inflammation [32, 65, 66, 67]. Moreover, we have recently found that PGE_2_ inhibits Treg cell expansion or accumulation in the intestine through T cell‐independent but microbiota‐dependent mechanisms [35]. The PGE_2_’s discrepant effects on Treg cells may be resulted from different settings of microenvironment, for example, under tumorous versus inflammatory conditions. It is worth to note that most of these findings were obtained from studies using in vitro cell culture systems, and nearly no such results were generated in vivo using genetically modified animals. In this current report, we have used global and T cell‐specific conditional EP4‐deficient mice to demonstrate the direct actions of PGE_2_ on suppression of iTreg differentiation in vitro and in vivo.

PGE_2_‐EP2/EP4‐cAMP signalling promotes Th1 and Th17 cells through inducing expression of IL‐12Rβ2 and IL‐23R, key cytokine receptors for Th1 and Th17 cell differentiation, respectively [24, 25]. Similarly, we found here that PGE_2_ inhibits iTreg cell development by reducing expression of TGF‐β receptors through EP2/EP4‐activated cAMP signalling, in which the downstream transcription factor CREB is possibly involved. CREB is important for TGF‐β‐induced Foxp3 transcription in T cells through binding of SMAD complex (i.e. SMAD2/3 and 4) to the CREB/CBP/P300 complex in the promoter region of Foxp3 gene [68]. Deficiency of CBP and p300 in Foxp3^+^ Treg cells impairs Treg cell stability and suppressive function, resulting in over‐activation of effector T cells and autoimmune inflammation [69]. CREB is also essential for TCR‐induced Foxp3 gene expression in vitro [70]. However, a recent research found that deficiency of CREB in T cells actually decreases Treg cell proliferation and survival and expands Th17 cell responses in vivo, resulting in exacerbation of T cell‐mediated autoimmune inflammation [71]. Thus, cAMP‐PKA‐CREB signalling may also contribute to PGE_2_ suppression of Ki‐67^+^Foxp3^+^ proliferating iTreg cells. Furthermore, the cAMP/PKA/CREB pathway has also been reported to antagonize the TGF‐β/SMADs pathway in multiple cell types [72]. Lack of TGF‐β or its receptors or interruption of TGF‐β/SMAD signalling prevents Treg cell development [73]. It is noteworthy that PGE_2_ also inhibited TGF‐β/IL‐6‐induced Th17 cell differentiation although it markedly upregulated IL‐23‐driven Th17 cell expansion [26]. Therefore, down‐regulation of TGF‐β receptors and upregulation of TGF‐β signalling inhibitors by PGE_2_ may collaboratively lead to diminished TGF‐β responsiveness during iTreg cell differentiation.

During iTreg cell differentiation, TCR engagement induces T‐cell activation and production of cytokines such as IL‐2 which through activation of the transcription factor STAT5 maintains or boosts Foxp3 expression [52]. Inhibition of STAT5 activity reduced Foxp3 expression during iTreg cell differentiation, which was further repressed by additional PGE_2_, excluding the possibility that PGE_2_ inhibits Foxp3 induction in mouse T cells through the IL‐2‐STAT5 pathway. However, our results indicate that interruption of IL‐2 signalling is likely involved in PGE_2_ suppression of human Foxp3 induction.

PGE_2_ signalling, especially through the EP4 receptor, is critical for T cell‐mediated chronic, autoimmune inflammation in numerous organs including skin, joint, brain and intestine [22]. This was considered to be mediated by promoting inflammatory Th1 and Th17 cells. Our findings in this report suggest that inhibition of Treg cells may be also an additional mechanism involved in PGE_2_ exacerbation of immune inflammation. EP4 deficiency‐increased Treg development and accumulation to inflammation cites in vivo, as observed in Figure 4, may also contribute, at least in part, to reduced T cell‐mediated inflammation in tissues such as intestine and skin [24, 26]. Although lack of EP4 alone in vivo is sufficient to increase Treg cell numbers in vivo, only blocking both EP2 and EP4 can rescue PGE_2_ suppression of iTreg induction in vitro and blocking either receptor had few effects. The differential requirement of EP2 on PGE_2_ suppression of iTreg cells in vitro and in vivo may arise from (1) divergent levels of EP2 and EP4 in vivo and/or (2) distinct binding capacity for PGE_2_ to EP2 and EP4 in vivo. Taken together, our findings suggest that therapeutically targeting PGE_2_‐EP4 signalling in T cells may be beneficial for treating immune‐mediated inflammation, partially due to modulation of Treg cells.

## CONFLICT OF INTEREST

The authors state no conflict of interest.

## AUTHOR CONTRIBUTIONS

MG, SC and CY designed and performed experiments and analysed the data. YZ analysed the GEO data sets. AGR provided intellectual input, reagents and edited the manuscript. SN and CY conceived this project and supervised the research. MG and CY wrote the manuscript with input from all authors.

## Data Availability

The data that support the findings of this study are available from the corresponding author upon reasonable request.
